# The Potential Cardiovascular Benefits of Physical Exercise in Early Onset Psychosis and Bipolar Disorder

**DOI:** 10.1192/j.eurpsy.2024.248

**Published:** 2024-08-27

**Authors:** H. C. Bohman, M. Lundberg

**Affiliations:** Neuroscience, Ki SÖS, Stockholm, Sweden

## Abstract

**Introduction:**

Early onset psychosis (EOP) and bipolar disorder (EOBP), occurring before the age of 18, have been linked to early signs of atherosclerosis and an elevated risk of cardiovascular disease (CVD). Physical exercise is a well-established factor in reducing the risk of developing CVD. However, it remains unclear whether regular physical activity can mitigate cardiovascular risk factors and signs of atherosclerosis in individuals with EOP and EOBP.

**Objectives:**

This study aimed to explore the impact of physical exercise on cardiovascular risk factors in these populations.

**Methods:**

We assessed the physical exercise habits of 71 individuals, including 22 with EOP, 21 with EOBP, and 28 age-matched healthy controls. Participants’ physical exercise routines were categorized as 0, 1, 2, or 3 or more times per week, with each session lasting at least 30 minutes. Our analysis included adjustments for conventional CVD confounders. Additionally, we used high-frequency ultrasound (22 MHz) to evaluate different layers of the arterial wall in the left common carotid artery (LCCA).

**Results:**

Compared to the control group, adolescents with EOP and EOBP exhibited significantly thicker LCCA intima thickness (0.132 vs. 0.095 mm, p<0.001) and intima/media ratio (0.24 vs. 0.17, p<0.001). Remarkably, adolescents with EOP and EOBP who engaged in physical exercise three times or more weekly (n=13) displayed significantly less intima thickness (0.142 vs. 0.116 mm, p<0.01). However, we did not observe a significant association between exercise and other CVD risk factors. Even when considering factors such as the extent of antipsychotic medication use or the severity of the disorders in our regression analysis, the significant association between exercise and reduced intima thickness persisted (p<0.05).

**Conclusions:**

Among adolescents with EOP or EOBP, those who engaged in physical exercise three or more times weekly exhibited less pronounced LCCA intima thickness compared to their less active counterparts, although it remained thicker than that of healthy controls. These findings, if replicated, suggest that regular physical exercise, specifically three or more times a week, could potentially offer protection against the future development of CVD in individuals with EOP and EOBP. Further research is warranted to confirm and expand upon these promising results.

**Disclosure of Interest:**

None Declared

